# Building the Foundation for International Conservation Planning for Breeding Ducks across the U.S. and Canadian Border

**DOI:** 10.1371/journal.pone.0116735

**Published:** 2015-02-25

**Authors:** Kevin E. Doherty, Jeffrey S. Evans, Johann Walker, James H. Devries, David W. Howerter

**Affiliations:** 1 United States Fish & Wildlife Service, 134 Union Blvd, Lakewood, Colorado, United States of America; 2 The Nature Conservancy, Fort Collins, CO, 80524, United States of America & Department of Zoology and Physiology, University of Wyoming, Laramie, WY, 82071, United States of America; 3 Ducks Unlimited, Great Plains Region, 2525 River Road, Bismarck, North Dakota, United States of America; 4 Institute for Wetland and Waterfowl Research, Ducks Unlimited Canada, P.O. Box 1160 Stonewall, Manitoba, Canada; Point Blue Conservation Science, UNITED STATES

## Abstract

We used publically available data on duck breeding distribution and recently compiled geospatial data on upland habitat and environmental conditions to develop a spatially explicit model of breeding duck populations across the entire Prairie Pothole Region (PPR). Our spatial population models were able to identify key areas for duck conservation across the PPR and predict between 62.1 – 79.1% (68.4% avg.) of the variation in duck counts by year from 2002 – 2010. The median difference in observed vs. predicted duck counts at a transect segment level was 4.6 ducks. Our models are the first seamless spatially explicit models of waterfowl abundance across the entire PPR and represent an initial step toward joint conservation planning between Prairie Pothole and Prairie Habitat Joint Ventures. Our work demonstrates that when spatial and temporal variation for highly mobile birds is incorporated into conservation planning it will likely increase the habitat area required to support defined population goals. A major goal of the current North American Waterfowl Management Plan and subsequent action plan is the linking of harvest and habitat management. We contend incorporation of spatial aspects will increase the likelihood of coherent joint harvest and habitat management decisions. Our results show at a minimum, it is possible to produce spatially explicit waterfowl abundance models that when summed across survey strata will produce similar strata level population estimates as the design-based Waterfowl Breeding Pair and Habitat Survey (r^2^ = 0.977). This is important because these design-based population estimates are currently used to set duck harvest regulations and to set duck population and habitat goals for the North American Waterfowl Management Plan. We hope this effort generates discussion on the important linkages between spatial and temporal variation in population size, and distribution relative to habitat quantity and quality when linking habitat and population goals across this important region.

## Introduction

Located in the interior of North America, the Prairie Pothole Region (PPR) is a unique wetland-grassland ecosystem [1] known for large populations of migratory birds including waterfowl [2,3], waterbirds [4,5], shorebirds [6], and grassland birds [7]. The PPR is named for the millions of depressional wetlands called “prairie potholes” dispersed throughout the landscape. The vast area of the PPR ecosystem and high density of wetland basins exceeding 40/km^2^ in some areas [8], makes the PPR region globally unique. Besides their critical importance to birds, remaining wetlands and grasslands in the PPR provide vital habitat for a diverse array of plant and animal species, including mammals [9], fishes [10], amphibians [11], and a variety of invertebrates [12]. This area also provides critical habitat for a number of threatened and endangered species.

Grasslands and wetlands within the PPR, especially the eastern PPR, are some of the most altered landscapes in the world because much of the land is privately owned, is productive as cropland, and is relatively easy to cultivate [13]. Conversion of the grasslands and wetlands for crop production continues [14–18]. Because of its vital importance to waterfowl and ongoing losses in both wetlands and grasslands, the North American Waterfowl Management Plan (NAWMP) identified the Prairie Pothole Region (PPR) as the continent’s top priority for waterfowl conservation. The Prairie Habitat Joint Venture (PHJV; Canadian PPR) and the Prairie Pothole Joint Venture (PPJV; U.S. PPR) were established by NAWMP in 1987 as 2 of the original 6 joint ventures to protect and restore critical waterfowl habitats [19] in this region. The PHJV and the PPJV are largely the same ecosystem, thus they share populations of migratory species whose populations fluctuate across the border depending on habitat conditions[18]. Lack of unified data layers and conservation planning tools has limited international conservation planning efforts between these groups in the past.

Development and evaluation of goals is fundamental in the adaptive management of wildlife populations and their habitat [20]. To evaluate the effectiveness of PHJV and PPJV efforts in conserving the habitat required to support waterfowl population goals, paradigms of population and habitat management must be linked. Historically, these paradigms have operated in isolation in part because of the regional and continental scales at which they have operated. Population management has generally addressed concerns about *how many*, while habitat management has generally concerned itself with issues of *how much* and *where*. Further, issues regarding *how often* habitat conditions will be conducive to waterfowl settling have emerged with respect to climate effects. These differences can confound management plans as well as the evaluation of those plans’ goals. For example, population goals derived from sample theory are largely an aspatial process generated from design-based surveys within a specific boundary such as a Joint Venture administrative boundary or a Bird Conservation Region [21]. Estimating population sizes and understanding population trends provides metrics to monitor wildlife populations and alert managers to species in need of conservation attention [22], thus surveys provide clear value. However, habitat losses [17,18], conservation planning [23,24], and ultimately delivery of habitat conservation programs to support bird populations [25] are inherently spatial processes which are conducted at finer scales than a JV or Bird Conservation Region Boundary. This disconnect in scale must be overcome to evaluate population goals from habitat management plans in light of habitat trends and local-scale habitat conservation decisions. The simplest first step towards integrating population and habitat goals is to understand the biological linkages between populations and habitat across broad spatial scales using data which is foundational to the management of the species. Subsequent steps can then involve estimating the interaction of local populations, habitats, and key vital rates in each region.

Further complicating evaluation of PPJV and PHJV habitat goals is climatic variability in both space and time. Climatic conditions in the PPR are characterized by substantial spatial and temporal variation in precipitation [26,27]. This variability influences the number of wetland basins containing ponded water each year (ponds), water levels within those ponds, and abundance of wetland-associated wildlife. Pond numbers and associated ecological functions vary during wet/dry precipitation cycles. Many species of wildlife are adapted to this variable environment and respond to wetland conditions and precipitation-driven grass conditions by changes in distribution and abundance [5,7]. Thus, the stability of the PPR system is largely achieved through its vast expanse and species that have evolved mechanisms (e.g., high vagility, drought tolerance) to cope with variable conditions over space and time. Periodic drying of wetlands maintains the productivity of prairie wetlands by accelerating nutrient cycling and allowing seeds of annual plants to germinate [28]. Consequently, environmental variation in the PPR drives ecosystem productivity and carrying capacity for migratory birds, but also complicates conservation planning because abundance and density of birds within localized landscapes fluctuate among years.

To facilitate joint conservation planning that accounts for broad ecosystem variability and change, and to evaluate the attainability of NAWMP population goals, the PPJV and PHJV need common planning tools. We used Waterfowl Breeding Pair and Habitat Survey data which is used to estimate the breeding population of ducks (BPOP) and recently developed geospatial data on upland habitat and environmental conditions to develop a spatially explicit model of breeding duck populations across the entire PPR. We had 3 main objectives for this effort: 1) Create a seamless spatially explicit conservation planning tool across the PPJV & PHJV which will allow us to bring international partners together to discuss coordinated actions and joint identification of key areas for duck conservation, 2) Generate a simple example of the importance of spatial heterogeneity and temporal variability in populations to generate discussion on importance of *how many, how often, how much*, and *where* in linking habitat and population goals at a continental scale, and, 3) Test if spatially explicit models of waterfowl distribution produce similar results to stratum-level population estimates. We felt the latter was important because stratum-level population estimates provide the basis for waterfowl harvest regulations and stepped-down population goals for individual Joint Ventures through NAWMP.

## Methods

### Study Area

**Fig 1:**
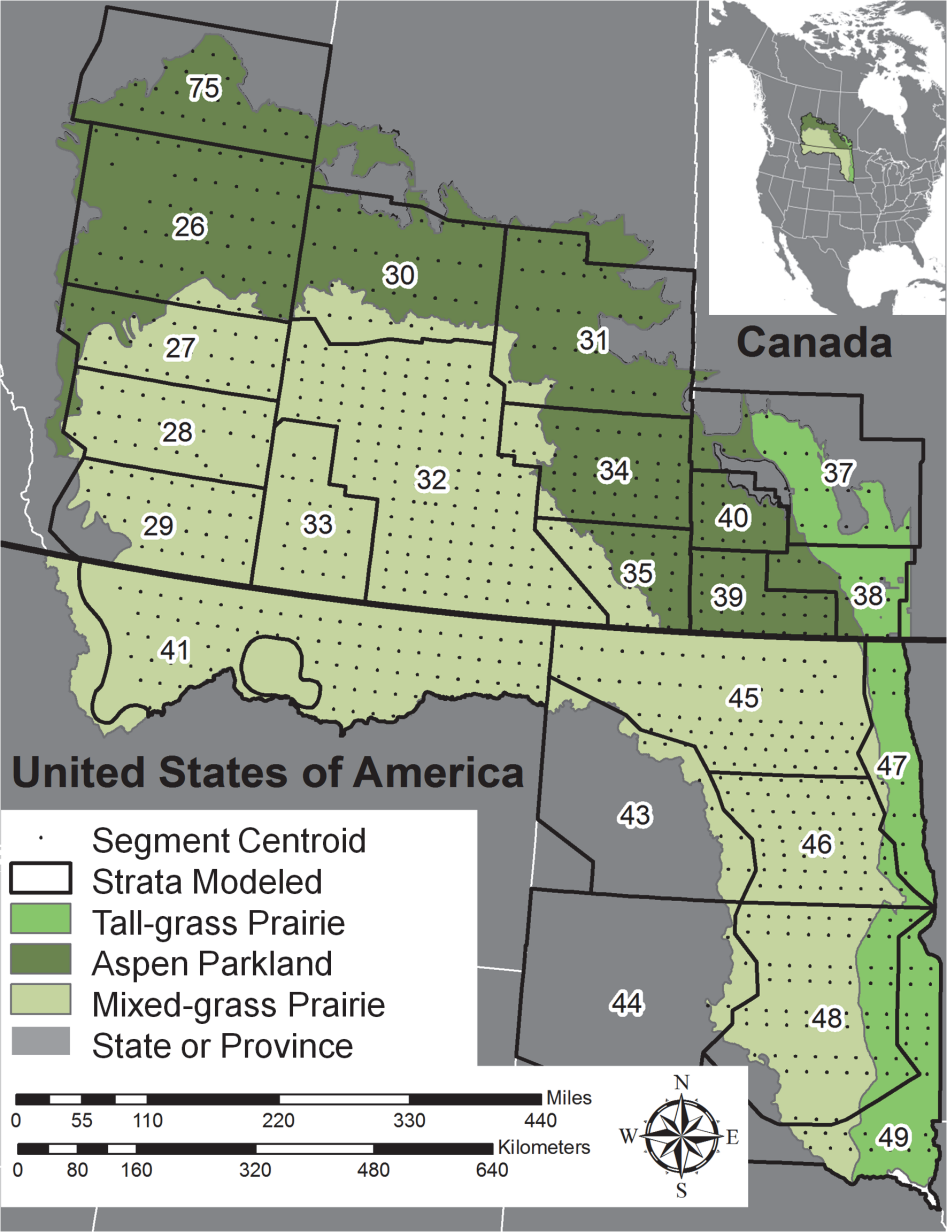
Location of the Prairie Pothole Region (PPR) of the U.S. and Canada. Ecoregions within the PPR are shown at their coarsest delineations to provide context for the settling patterns of 5 species of dabbling ducks across the traditional Waterfowl Breeding Population and Habitat Survey (BPOP) areas in the Prairie Pothole Region during 2002–2010. These species included; blue-winged teal (*Anas discors*), gadwall (*A. strepera*), mallard (*A. platyrhynchos*), northern pintail (*A. acuta*), and northern shoveler (*A. clypeata*). In this pilot effort, we only modeled areas within the traditional BPOP survey area within the PPJV & PHJV boundaries. Stratum boundaries and transect centroids show the spatial distribution of the BPOP survey population data which was linked to GIS based habitat variables.

The PPR straddles the U.S./Canada border and encompasses > 770,000 km^2^ (>297,000 square miles) including parts of 5 U.S. states: northern Montana, northern and eastern North Dakota, eastern South Dakota, western Minnesota, and north-central Iowa; and 3 Canadian provinces: southwestern Manitoba, southern Saskatchewan and southern Alberta. For this pilot project we restricted our analysis to that portion of the PPR that falls within the BPOP traditional survey area (Fig. 1).

### Waterfowl Population Data

Breeding ducks have been counted along aerial transects annually since 1955. This survey is the longest-running dataset on the status of any group of wildlife species in the world [1] and has produced insight into both ecology [29] and wildlife management [30]. The traditional survey covers approximately 3.4 million km^2^ with transects flown throughout most of the duck breeding habitat in the northern United States and much of Canada [1]. Information from this survey is the primary consideration when hunting regulations are established through the international regulatory process within the flyway council system. The total BPOP survey area is split into strata, which are the units at which individual duck species populations are estimated by the USFWS Division of Migratory Bird Management (Fig. 1). Within strata, numerous systematically spaced east-west transects are flown with fixed-wing aircraft at 30–45 m above ground level and all ducks within 200 meters on both sides of transects are counted by the pilot and an aerial observer [31]. Each transect is further divided into segments that average approximately 29-km in length (∼18 miles; Fig. 1). Aerial counts are compared to simultaneous ground counts on a subset of survey segments within each strata to estimate detection probability for each stratum [31]. We modeled total pairs of ducks counted within a segment to develop our spatially explicit statistical models of the abundance of breeding ducks across the PPR. Total pairs included both indicated pairs (i.e. isolated lone drakes) and pairs (both male and female in close proximity). We corrected counts for detection probability by multiplying total counted pairs within a segment by the corresponding stratum level visibility correction factor (VCF). Only segments entirely within the PPR boundary with GIS-based habitat coverage were included in our analysis. Duck counts are publicly available and were downloaded from the USFWS Division of Migratory Bird Management Migratory Bird Data Center [32].

Our response variable was the sum of 5 species of dabbling duck pairs within each segment within each year from 2002–2010. These species included; blue-winged teal (*Anas discors*), gadwall (*A. strepera*), mallard (*A. platyrhynchos*), northern pintail (*A. acuta*), and northern shoveler (*A. clypeata*). We chose these species because they are the most abundant and widely distributed breeding duck species in the PPR. We chose to lump these species because existing waterfowl conservation planning tools within the PPJV and PHJV use the sum of these 5 species as the primary determinant of waterfowl conservation priority areas. The abundance and wide distribution of these species across the PPR makes them suitable for modeling species-habitat relationships because we were able to sample different population densities across a wide range of habitat conditions in the PPR. This minimizes the probability of encountering novel landscape conditions and their associated challenges in habitat modeling [33,34].

The spatial extent at which we modeled abundances (i.e. the entire PPR), and the resolution at which we modeled population response to habitat selection (11 km^2^ scale) were chosen to match our study objectives. Our modeling efforts represent an intermediate level of habitat selection between the geographic range of the species in our analysis and individual home ranges [35]. This level of selection demonstrates variation in population density across broad regional scales [35,36]. We chose this scale because our goal was to develop an ecosystem-wide model and it closely matches regional scales at which habitat programs are delivered in the PPJV and PHJV.

### Predictor Variables

We selected predictor variables based on their demonstrated or hypothesized linkage to abundance and distribution of the study species. We calculated values of each predictor within the ∼11 km^2^ survey segment using publicly available geospatial data and standard tools in ArcGIS software. Native pixel resolution varied amoung predictor variables (Table 1), however in all cases we characterized habitat at a ∼ 11 km^2^ scale. We did this by up or down-sampling all rasters so they aligned with our 400 m^2^ sample grid. We then used a moving window to characterize habitat conditions at a ∼ 11 km^2^ scale area around each sample grid. Predictor variables described wetland characteristics, drought status, topography, climate, landuse-landcover, and primary productivity of vegetation across the study area. We separated predictors into 2 groups: 1) predictors with established relationships to abundance and distribution of breeding ducks or wetlands containing ponded water, which is a strong correlate of breeding duck abundance, in the published literature (hereafter, established predictors) and 2) predictors that we hypothesized to have relationships with abundance and distribution of breeding ducks or wetlands containing ponded water but for which little or no direct published evidence was available (hereafter, exploratory predictors).

**Table 1:** Description of the explanatory variables used to predict the abundance of count of 5 species of dabbling ducks within a ∼ 11 km2 scale within the Prairie Pothole Region of the U.S. and Canada during 2002–2010.

Predictor group	Name	Abbreviation	Source (years)	Native resolution	Resampled resolution	Description	Justification [references]
Established	Wetland count	NA	NWI (1985), CanVec, DUC	Polygon	0.16 km^2^	number of wetland basins	Established positive relationship between breeding duck abundance and pond abundance [3,41,42]
	Wetland area	NA	NWI (1985), Can Vec, DUC	Polygon	0.16 km^2^	total area of wetland basins	Established positive relationship between breeding duck abundance and pond abundance [3,41,42]
	Ponds	Ponds_t, t-1, t-2_	BPOP (2002–2009)	11 km^2^	0.16 km^2^	year-specific number of wetland basins containing ponded water	Established positive relationship between breeding duck abundance and pond abundance in the PPR [3,41,42]
	Normalized Difference Wetness Index	NDWI_t,_	NASA EODP MODIS (2002–2009)	1.00 km^2^	0.16 km^2^	year-specific hydrologic state of wetland basins, soil, and vegetation	Established positive relationship between pond abundance and precipitation [12,27–29]
	Palmer Drought Severity Index	PDSI _t-1, t-2_	NIDIS	30,625 km^2^	0.16 km^2^	year-specific drought status	Established negative relationship between pond abundance and drought [12,27–29]
Exploratory	Topographic variation	SRTM_CV	NASA SRTM	0.009 km^1^	0.16 km^2^	variation in elevation	Hypothesized positive relationship between breeding duck abundance and reproductive success and topographic variation
	Degree days greater than 5 C	DD5	USFS (1961–1990) [48]	1.00 km^2^	0.16 km^2^	degree days greater than 5 C	Hypothesized relationship between breeding duck abundance and reproductive success and land use mediated by climate [18,46,47]
	Annual moisture index	AMI	USFS (1961–1990) [48]	1.00 km^2^	0.16 km^2^	ratio of degree days greater than 5 C to mean annual precipitation	Hypothesized relationship between breeding duck abundance and reproductive success, land use, and wetland habitat mediated by climate [18,46,47,49,50]
	Summer-spring precipitation balance	SSPB	USFS (1961–1990) [48]	1.00 km^2^	0.16 km^2^	ratio of summer to spring precipitation	Hypothesized relationship between breeding duck abundance and reproductive success and wetland habitat mediated by climate [49,50]
	Proportion cropland	Crop	NLCD, AAFC	0.009 km^0^	0.16 km^2^	proportion of the landscape composed of cropland vegetation types	Hypothesized negative relationship between proportion cropland and breeding duck abundance and reproductive success [12,47,52,53]
	Proportion grassland	Grass	NLCD, AAFC	0.009 km^1^	0.16 km^2^	proportion of the landscape composed of grassland vegetation types	Hypothesized positive relationship between proportion grassland and breeding duck abundance and reproductive success [12,47,52,53]
	Proportion forest	Forest	NLCD, AAFC	0.009 km^2^	0.16 km^2^	proportion of the landscape composed of forest vegetation types	Hypothesized negative relationship between proportion forest and breeding duck abundance and reproductive success [12,47,52,53]
	Gross primary productivity	GPP _t-1, t-2_	MODIS NASA EODP (2002–2009)	1.00 km^2^	0.16 km^2^	year-specific maximum gross primary productivity during April-July nesting season	Hypothesized positive (or negative) relationship between reproductive success and recent GPP [41]

These species included; blue-winged teal (Anas discors), gadwall (A. strepera), mallard (A. platyrhynchos), northern pintail (A. acuta), and northern shoveler (A. clypeata).

Footnote: data source abbreviations in order of appearance: National Wetlands Inventory (NWI); (CanVec); Ducks Unlimited Canada (DUC); United States Fish and Wildlife Service and Canadian Wildlife Service Breeding Population Survey (BPOP); National Aeronautics and Space Administration (NASA), Earth Observation Data Portal (EODP), Moderate Resolution Imaging Spectrometer (MODIS); Shuttle Radar Topography Mission (SRTM); National Landcover Dataset (NLCD), Agriculture Agri-Food Canada (AAFC). All data layers are available from https://www.sciencebase.gov/catalog/item/535fa1aae4b078dca33ae3ad?community=LC+MAP+-+Landscape+Conservation+Management+and+Analysis+Portal.

### Established Predictors

We designated survey segments according to whether they were located in the US or Canada to account for between-country differences in baseline wetland information (i.e., basin area and basin count) [37]. In the US, wetland variables were derived from the National Wetlands Inventory (NWI), a comprehensive digital archive of wetland polygons derived from digitized 1-m aerial photography [38]. In Canada, wetland variables were derived from a publicly available CanVec wetlands layer created from best available sources ranging in scale from 1:10,000 to 1:50,000 [39]. The CanVec wetlands were corrected using a spatial model built with Ducks Unlimited Canada's wetland inventory data (digitized wetlands at a scale of 1:5000 or better (imagery resolution 0.5 m—2.5 m), and Soil Landscapes of Canada [40]. Exact methods for integrating wetlands data between countries are detailed in Ryba et al. (2012).

We used 3 variables to describe spatial and temporal variation in wetland habitat. The first 2 variables were temporally static and reflected the number and total area of wetland basins on each segment [37]. The third wetland variable was temporally dynamic and was calculated as the number of wetland basins containing ponded water (ponds) within a segment each year during the May BPOP survey [32]. We corrected aerial pond counts using stratum level visibility correction factors using the exact methods we corrected duck count data for detection [32]. We also included information about pond numbers in the previous two years (t-1 and t-2) to account for a potential time-lagged effect of reproductive success in past years on current-year abundance [41]. May pond counts each year at the segment level were extrapolated to an ecosystem-wide GIS layer using an inverse-distance-weighted function in the geospatial modeling tool in ArcGIS 10.0. It is known that waterfowl populations respond to pond counts across the PPR [3]. We hypothesized that landscapes with greater numbers and area of wetland basins would have greater overall carrying capacity for breeding ducks [42] and potentially be associated with greater abundance and reproductive success during the initial years of wet periods.

We included 2 additional variables that measured moisture on the landscape because of the known importance of wetlands to ducks. These variables could potentially capture variation in duck abundance that related to potential current and time-lagged effects of wet-dry cycles on wetlands, soil, and vegetation. These variables were included as additional potential predictors and biologically were included for the same reason as our three other wetland variables. First, we included the Normalized Difference Wetness Index (NDWI) which is a measure of surface reflectivity and described combined surface water, soil moisture and water content of vegetation. NDWI is a derivative of Moderate Resolution Imaging Spectrometer (MODIS) [43] and was calculated as the NDWI value measured the closest to May 15th each year. Second, we used Palmer’s Drought Severity Index (PDSI), a standardized and widely accepted index of monthly moisture regime. We used global 2.5° (∼175 km) gridded monthly PDSI data for May [44]. We interpolated gridded values across the PPR in ArcGIS using inverse distance weighting and estimated segment-specific PDSI values using focal mean pixel values within survey segment boundaries. Because the effect of wet years may have carryover effects in subsequent years, we included one and two year lags for PDSI (t-1, t-2).

### Exploratory Predictors

We expected topography to affect the permanence of wetland basins and land use (i.e., more permanent basins, and more grass-based agriculture in rolling topography; more seasonal basins, and more crop-based agriculture in flatter topography). We used Shuttle Radar Topography Mission (SRTM3 Version 2; 3 arc-second resolution ∼90 m) digital elevation model data to characterize landform within survey segment boundaries [45]. We first used Spatial Analyst in ArcGIS 10.0 to generate a surface representing the coefficient of variation (CV) in elevations within a 41 km^2^ neighborhood. We subsequently estimated our landform covariate (SRTM_CV) as the focal mean of elevation CVs within survey segment boundaries.

We expected climate variables, which were spatially explicit long-term averages, to be important because climate constrains land use, affects wetland dynamics, and drives ecosystem composition and dynamics. Rate and extent of conversion of grasslands varies across the PPR’s sub-regional climate gradients. For example, grassland losses are greater within the tall-grass ecosystem [18]. Tall-grass prairies occur at the higher end of the precipitation gradient of global grasslands and agricultural conversion has almost extirpated this vegetation community [46]. Demographic rates are also known to vary among climatic gradients within the PPR [47]. We therefore included 3 environmental variables related to broad-scale climatic patterns for all of North America during 1961–1990 [48]. Climate variables were highly correlated, so we chose variables that were most relevant to our hypotheses and had correlations ≤ 0.65. We included the number of degree-days > 5^°C^ (DD5). We also included an annual moisture index (AMI) which was calculated by dividing DD5 by the mean annual precipitation. Because of the importance of wetlands retaining water to nesting effort and brood survival [49, 50], we included the summer/spring precipitation balance (SSPB) as an index of how long the temporary and seasonal wetlands within the PPR were expected to remain wet on average [49,50].

We used 3 variables to describe landuse-landcover at the segment level: proportion of cropland, proportion of grassland, and proportion of forest. These variables were calculated from 30-m landcover products created in 2001 by Agriculture Agri Food Canada and the United States Geological Survey National Land Cover Dataset program [51]. We calculated values of these variables for each segment in the dataset. Values of the landuse-landcover variables did not vary among years. We hypothesized that abundance, distribution and demographic rates of ducks would be related to landuse-landcover. For example, nest survival probability [47,52] has been shown to be positively related to the proportion of grassland in the landscape. Nest survival probability has also been shown to be negatively related to the proportion of cropland in the landscape [53]. These patterns could result from both bottom-up and top-down effects. For example, high-protein invertebrate food resources critical for egg formation and duckling growth tend to be less abundant in wetlands surrounded by cropland [12]. As a top down example, predator communities tend to be more diverse and include an avian component in landscapes with more forest cover, thus nest survival tends to be lower in these landscapes [53].

We included a year-specific measurement of maximum Gross Primary Productivity (GPP) on each segment during the two previous years (t-1 and t-2). GPP provided an index to the amount of vegetation growth on a given site and year [54]. GPP in previous years was associated with nest success, such that populations nest success was higher with greater GPP in the prior year, but lower with greater GPP two years prior [41]. If natal philopatry is evident at this scale, GPP may be predictor of population settling. GPP is derived from MODIS satellite imagery and collected at 8-day intervals. We obtained MODIS from the National Aeronautics and Space Administration’s Earth Observations Data Portal. GPP was calculated as the maximum GPP measurements during April-July on the 1-km^2^ MODIS pixel nearest the center of the transect centroid.

### Statistical Methodology

Statistical analysis was conducted in the statistical software R [55]. Data preparation was conducted using the rgdal [56], sp [57] and raster [58] libraries to read spatial data, assign values from spatial covariates to the point observations of our dependent variable and make spatial predictions that can be incorporated into any GIS environment. We used the nonparametric model Random Forests [59] implemented in the R library randomForest [60]. Random Forest is a bootstrapped Classification and Regression Tree (CART) approach that is based on the principle of weak learning [61]; where a set of weak subsample models converge on a stable global model. This method has been shown to provide stable estimates while being robust to many of the issues associated with spatial data (e.g., autocorrelation, nonstationarity). It also fits complex, nonlinear relationships and accounts for high dimensional interactions [62,63]. First and second order variation are addressed in the hierarchal nature in the iterative node partitioning making this a good model to implement when global trend and local variation [64] are expected to occur in the same model [63]. We expected both global trends in duck settling patterns as well as localized population clusters across the PPR based on prior models built within the U.S. PPR [25], the Canadian PPR [24], and discussions with USFWS pilots who fly the surveys. We followed the model selection method introduced in Murphy et al. (2010) using R code provided by those authors. Parsimony in Random Forests is important not only for producing a more interpretable model but also for reducing overall noise, thus providing a better model fit [63,65]. Nonparametric methods are becoming much more common in ecological modeling, supporting inference of nonlinear and spatial dynamics [62,63,66,67]. Random Forest modeling uses a bootstrap approach that tests a null distribution against the selected model is a robust way to test model significance in nonparametric models and has been previously published [63,65]. Inference was supported by following methods presented in Evans et al. (2011), Murphy et al. (2010) and Cutler et al. (2007). Given the expected complexity in variable interaction, potential latent variables, high spatial variability representing both global and local effects and nonlinear relationships, we felt a non-linear model such as Random Forest was an appropriate choice.

We produced year-specific models to fulfill our objective to describe temporal variation in the spatial distribution of waterfowl. These year-specific models also allowed us to compare our estimates to BPOP estimates at a stratum level. We used our annual predictive models to generate mean predictions and measures of variation through a two-stage modeling process [68]. Our logic is analogous to statistical methods that create habitat selection models for individual animals and then average across individuals to produce population level habitat selection inference [69]. In our analyses the first stage was each year specific model and our second stage was the averaged (or SD) abundance through time. Using two-stage processes are also a relatively simple way to allow heterogeneity in year-specific habitat selection to be incorporated into our spatially explicit model. This is important if it is expected that functional response relationships may change with variation in habitat condition through time. Two-stage processes also minimize issues of correlations between landscape conditions at sample points through adjacent years. Because we applied our statistical model back to each grid cell on the entire PPR landscape each year, we could readily calculate population metrics from our grid surfaces such as mean, max, and measures of variability such as standard deviations.

### Spatial Heterogeneity and Variability

**Fig 2:**
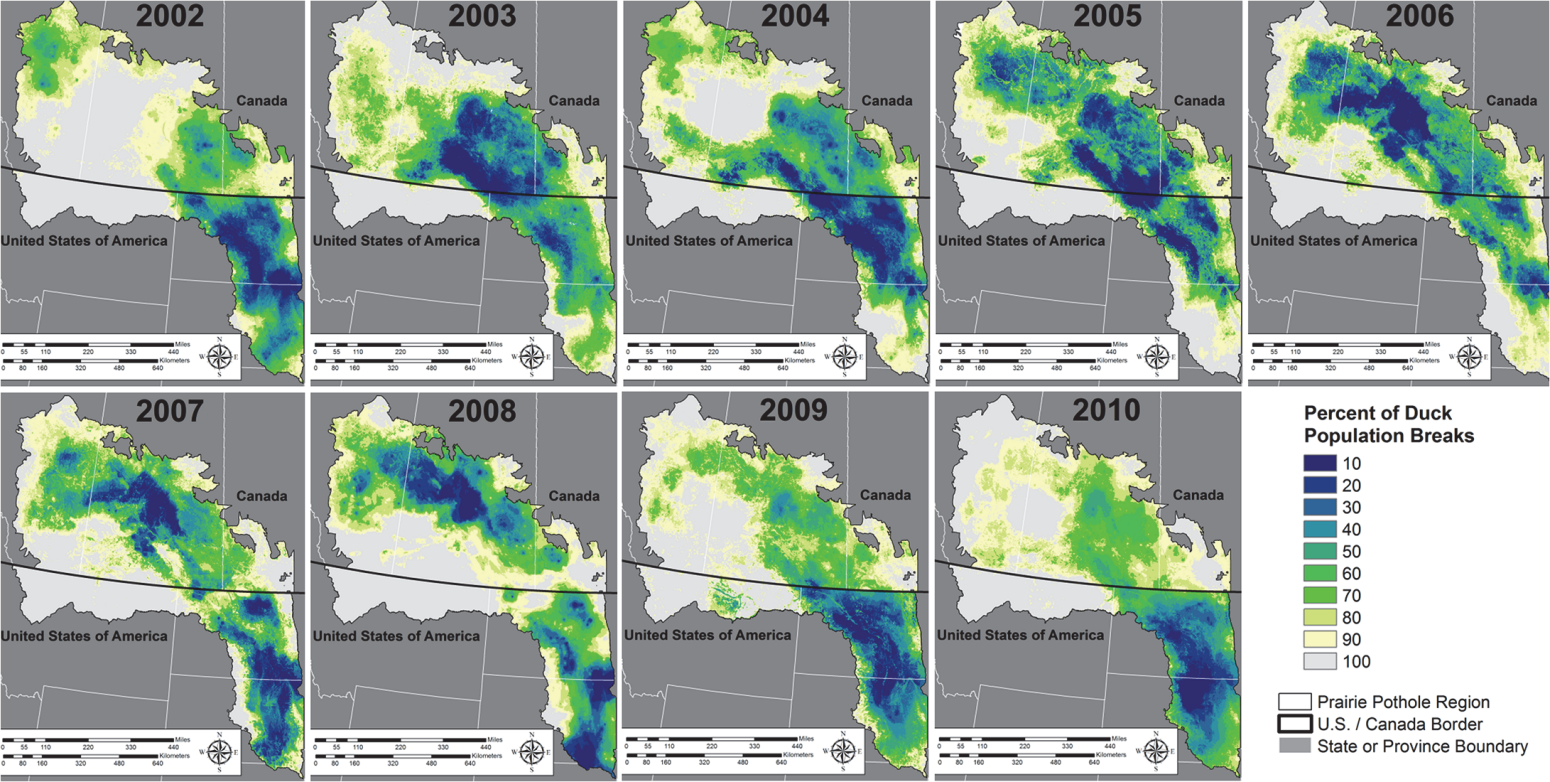
Abundance and distribution of 5 species of dabbling ducks across the traditional BPOP survey areas in the Prairie Pothole Region during 2002–2010. These species included; blue-winged teal (*Anas discors*), gadwall (*A. strepera*), mallard (*A. platyrhynchos*), northern pintail (*A. acuta*), and northern shoveler (*A. clypeata*). Population estimates derived from our spatially explicit models were summed across the entire landscape and grouped into 10 percent bins, such that a value of 10 represents the smallest area in which 10% of the population is contained relative to each year. Our spatially explicit population estimates show large variation in both population estimates and settling patterns across the years we modeled. Models explained between 64% and 79% of the variation in population counts.

We generated two map products highlighting the importance of spatial and temporal variability in population distributions to harvest management and conservation planning. First, we illustrated how variability in space is an important component in conservation planning which could require redundancy in habitat areas to support desired population goals because we know spatial distribution of birds vary in the PPR. We demonstrated this by showing the spatial variability in waterfowl abundance each year from 2002–2010 and calculating the SD and the maximum abundance for each grid cell. We processed duck density predictions each year and turned predicted density values into a relative percent of the PPR population. We defined the PPR population as the sum of all predicted duck counts from all grid cells. We then placed each grid cell in context of the PPR population by dividing the predicted grid cell density by the year-specific PPR population prediction. Starting with the highest density, we cumulatively summed the number of ducks predicted until each 10% percent population threshold was met. This resulted in a defined percent of the duck population being identified in areas of the highest density of breeding sites through each year (Fig. 2).

Second, we compared the two closest VCF corrected total pond counts during the years 2002–2010. We did this to assess if the most similar pond counts in our study would produce different spatial patterns across the landscape of the PPR. We did this because pond counts are the primary habitat predictor variable used in setting waterfowl harvest regulation.

### Comparison of spatially explicit model estimates versus BPOP estimates

We rescaled our grid surface predictions to account for the different size between our response variable (predicted number of ducks within ∼11km^2^) and our 400m^2^ grid cells. We then summed our grid surfaces to generate population estimates within strata that were fully contained within our study area. Within each stratum we then multiplied our estimates by 2 because we modeled pairs of ducks and BPOP estimates total individual ducks. We compared predictions graphically and by regressing the sum of the random forest grid cell predictions as the predictor of BPOP estimates within a stratum. Comparison of results generated from a design-based estimator to results from random forest modeling serves as evaluation through concurrence.

## Results

**Fig 3:**
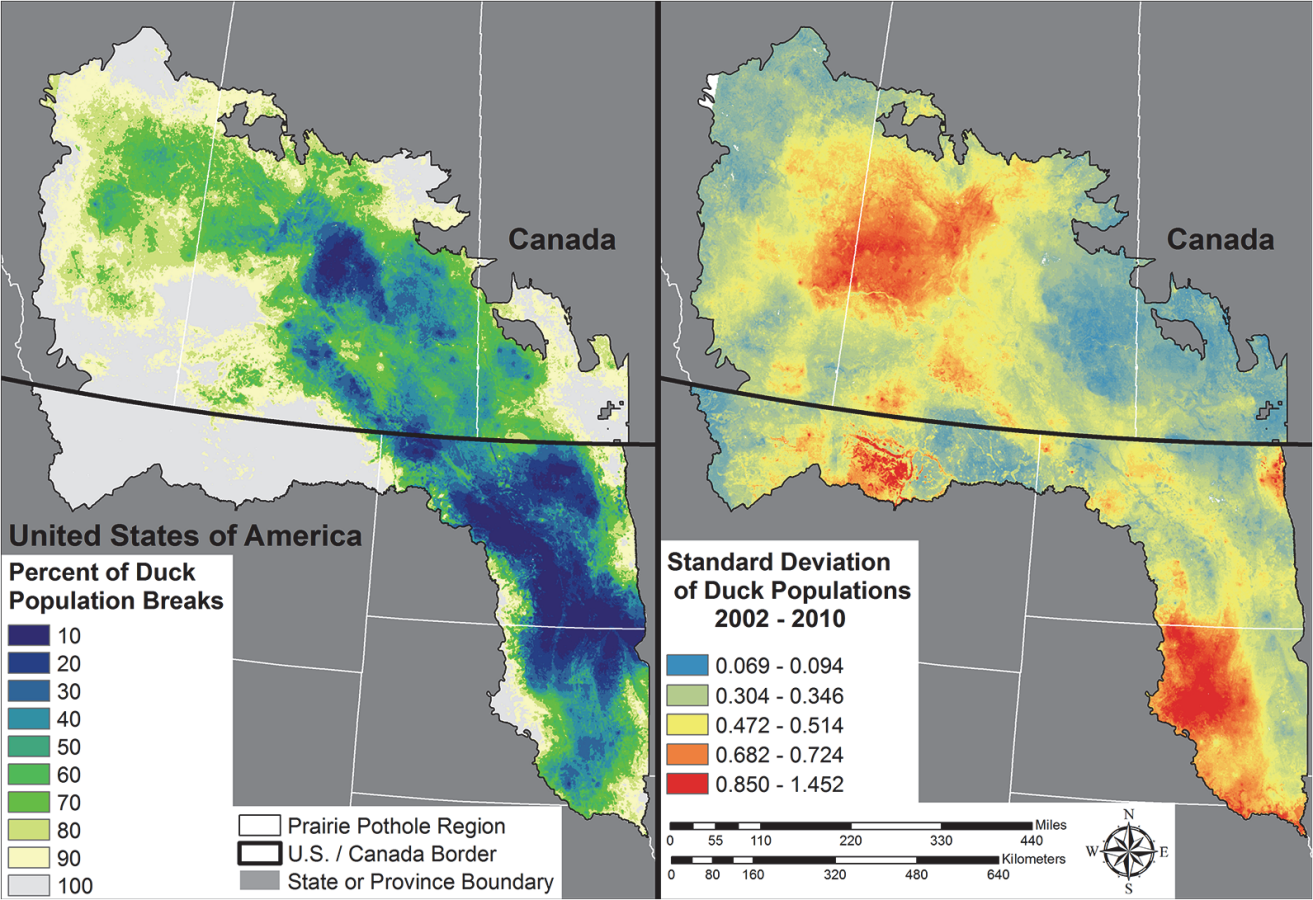
Abundance and distribution of 5 species of dabbling ducks across the U.S. and Canadian Prairie Pothole Region. These species included; blue-winged teal (*Anas discors*), gadwall (*A. strepera*), mallard (*A. platyrhynchos*), northern pintail (*A. acuta*), and northern shoveler (*A. clypeata*). Maps depict the mean and standard deviation of our yearly predictions from 2002–2010. For the mean population estimate (left inset) estimates were summed across the entire landscape and grouped into 10 percent bins, such that a value of 10 represents the smallest area in which 10% of the population is contained relative to each year.

On an annual basis duck populations clearly fluctuated around the PPR, which highlighted the importance of spatial heterogeneity and variability in these populations (Fig. 2, Fig. 3). We explained 68.4% of the variation in the counts of ducks on average within a transect segment during 2002–2010. Percent variation explained ranged from a low of 62.1% in 2005 to a high of 79.1% in 2010 (Table 2). Each year, 20 variables were offered as potential predictors of duck abundance. On average, model selection retained 12.4 predictors across years (Table 2). Number of visibility corrected ponds counted concurrently with the duck count was consistently selected as the strongest variable in each of the 9 years modeled and was always positively associated with settling (Table 3). Pond conditions during *t* – 1 & *t* – 2, AMI, PDSI *t*–1 & *t*–2, DD5, and wetland area were all consistently in the top 5 variables predicting waterfowl abundance (Table 3). GPP was included in 3 of the 9 years modeled, whereas NDWI was only retained as a predictor in 1 of the 9 years modeled. Comparing predicted duck counts at the segment level to hold-out samples of the data from each bootstrap replicate indicated good model fit (RMSE avg. = 0.7, range 0.3–0.9, Table 4). The median difference between observed and predicted ducks was 4.6, with an average difference of 0.5 ducks (Table 4). To put these differences in context, across the entire modeled area and years, the average count of the 5 species of ducks at a transect segment level was 117.3, with a median count of 77.4.

**Table 2:** Variation explained by year and the number of predictor variables selected by Random Forest model selection techniques.

Year	Percent Variance Explained	Number of Variables
2002	74.7%	10
2003	63.8%	16
2004	68.1%	11
2005	62.1%	16
2006	63.2%	11
2007	64.2%	11
2008	65.0%	10
2009	75.1%	16
2010	79.1%	11
Avg.	68.4%	12.4

**Table 3:** Top 5 variables selected for each year from 2002–2010.

Year	1^st^ Variable	2^nd^ Variable	3^rd^ Variable	4^th^ Variable	5^th^ Variable
2002	Pond _*t*_ (1.00)	Pond _*t-1*_ (0.77)	Pond _*t—2*_ (0.63)	PDSI _*t—2*_ (0.43)	PDSI _*t-1*_ (0.42)
2003	Pond _*t*_ (1.00)	PDSI _*t-2*_ (0.35)	AMI (0.34)	Pond _*t—2*_ (0.33)	DD5 (0.27)
2004	Pond _*t*_ (1.00)	Pond _*t-2*_ (0.69)	Pond _*t-1*_ (0.61)	PDSI _*t-1*_ (0.48)	Wetland Area (0.42)
2005	Pond _*t*_ (1.00)	Pond _*t-2*_ (0.80)	AMI (0.59)	Forest (0.58)	Pond _*t-1*_ (0.50)
2006	Pond _*t*_ (1.00)	AMI (0.67)	Forest (0.42)	Pond _*t-1*_ (0.39)	Pond _*t-2*_ (0.34)
2007	Pond _*t*_ (1.00)	AMI (0.95)	Pond _*t-1*_ (0.55)	PDSI _*t-1*_ (0.50)	Wetland Area (0.49)
2008	Pond _*t*_ (1.00)	AMI (0.67)	PDSI _*t-2*_ (0.64)	Pond _*t-2*_ (0.45)	Pond _*t-1*_ (0.44)
2009	Pond _*t*_ (1.00)	DD5 (0.53)	Wetland Area (0.46)	Country (0.43)	Wetland Count (0.35)
2010	Pond _*t*_ (1.00)	PDSI _*t-1*_ (0.60)	Pond _*t-1*_ (0.49)	DD5 (0.48)	AMI (0.43)

Variables importance values are scaled each year so that the top variable equals 1 and the remaining variables are a proportion derived by dividing by the top variable. They are derived from probability scaled partial plots in the RandomForest package in R. Wetland count and area are derived from GIS based polygon layers [37]. Pond Count derived from inverse distance weighting of aerial pond counts [32].

**Table 4:** Goodness of fit statistics generated from comparing model predictions versus the out of bag test data.

Year	RMSE	Min	1^st^ Quartile	Median	Mean	3^rd^ Quartile	Max
2002	0.3	-222.0	-5.3	2.5	0.1	9.7	73.1
2003	0.8	-186.9	-8.3	4.6	0.6	14.2	90.1
2004	0.6	-112.1	-8.5	4.3	0.4	11.9	61.2
2005	0.9	-149.8	-7.0	4.3	0.8	13.1	59.7
2006	0.8	-174.0	-9.3	4.5	0.6	15.7	88.6
2007	0.8	-236.6	-10.8	5.7	0.6	18.7	88.2
2008	0.8	-198.3	-8.5	5.2	0.6	16.2	70.9
2009	0.8	-184.4	-9.7	6.2	0.7	16.7	103.8
2010	0.7	-190.3	-8.1	3.8	0.5	13.9	91.2
Avg.	0.7	-183.8	-8.4	4.6	0.5	14.4	80.7

Metrics are computed by subtracting the observed duck counts from the predicted model counts.

**Fig 4:**
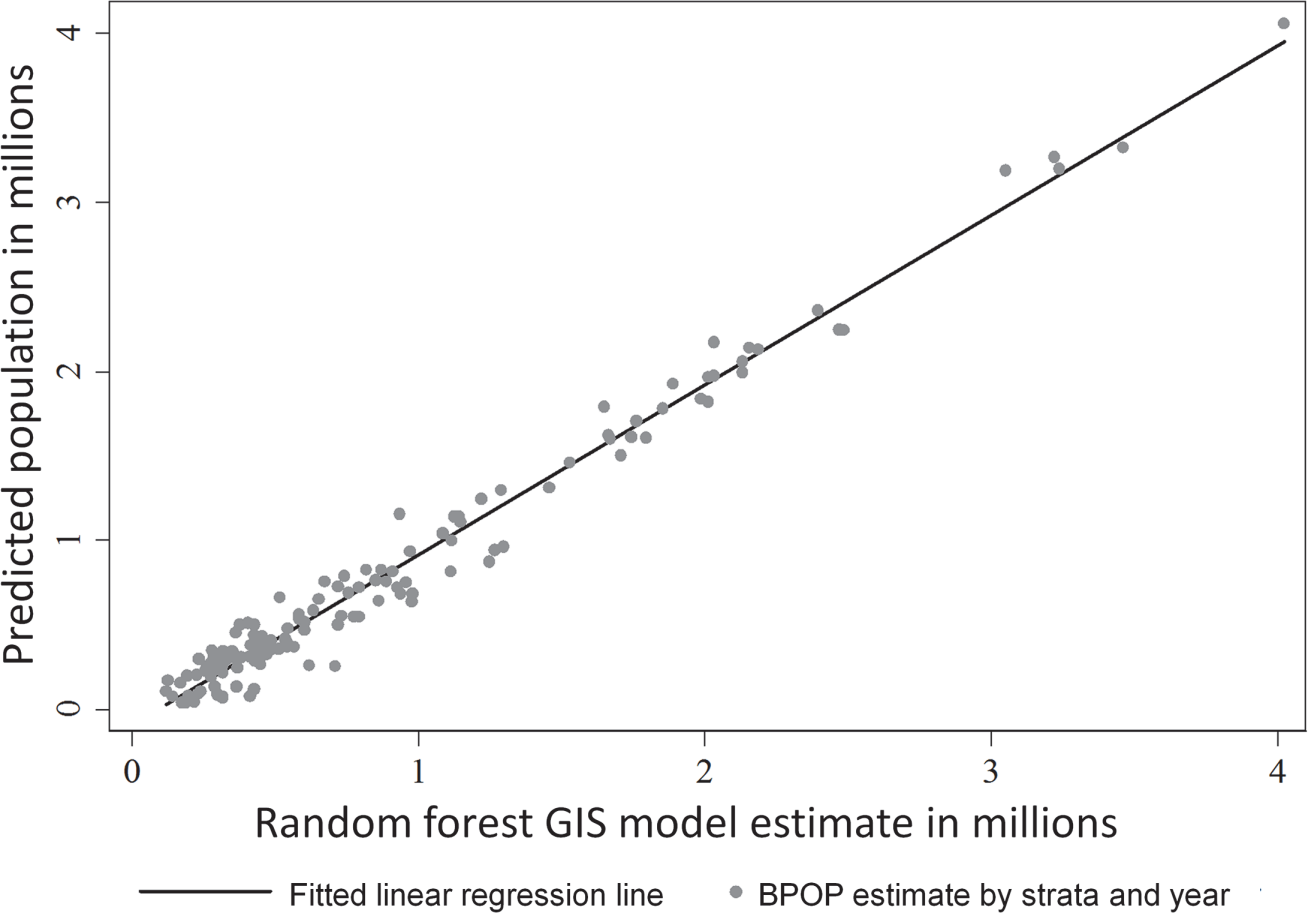
Linear regression of mean year and stratum level BPOP estimates as predicted by compared random forest stratum level population estimates from 2002–2010. Random forest estimates predicted BPOP estimates well with an r^2^ = 0.977 and a regression coefficient of 1.005. Plots of BPOP estimates versus random forest predictions highlight a good model fit, but also show variation for certain transect and year combinations.

**Fig 5:**
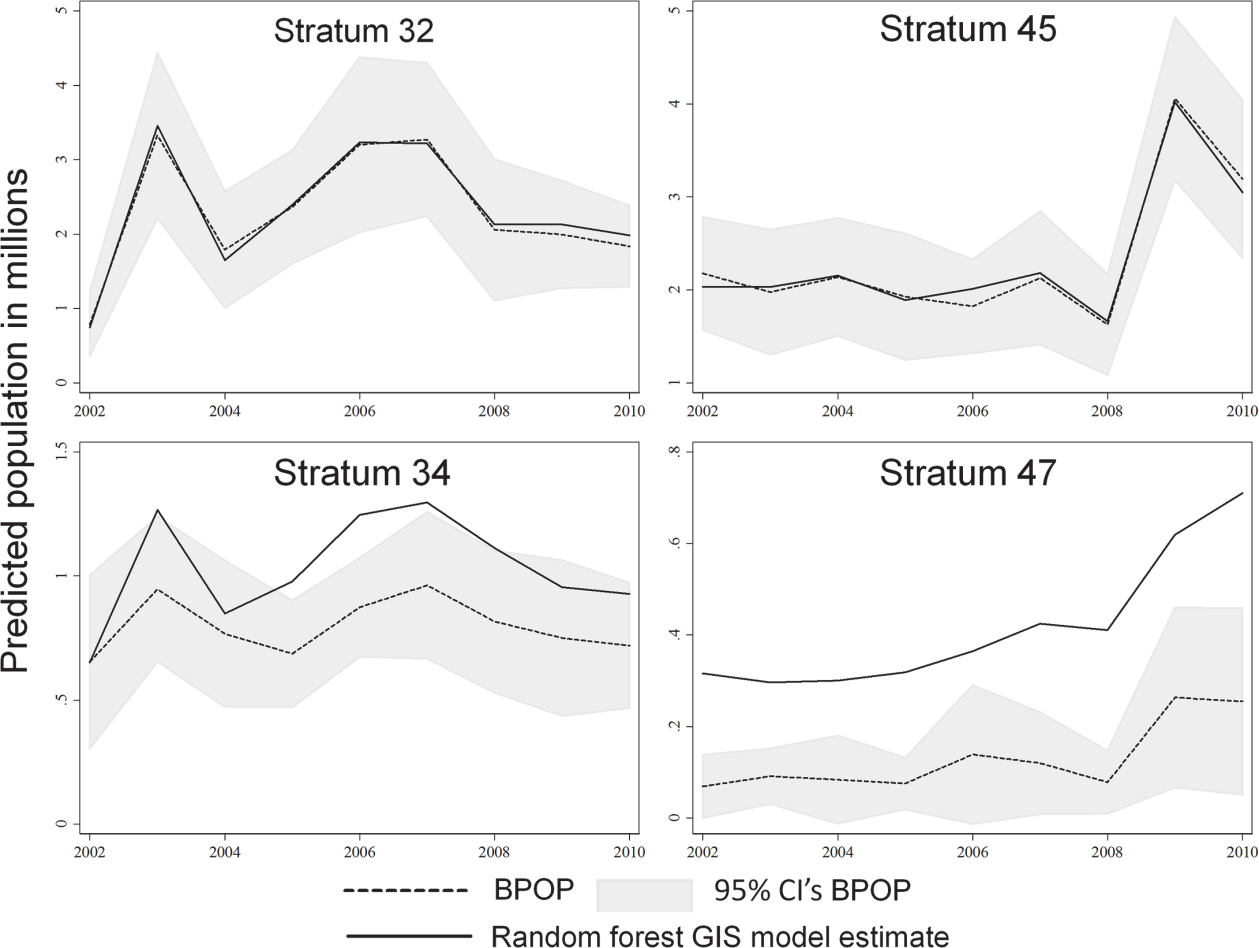
BPOP population estimates and random forest population estimates track each other well in most population strata and in the U.S. (45) and Canada (32) strata that have the highest ducks during 2000–2010. However, strata 34 & 47 were the two strata that consistently had highest standardized residual < -2. Post hoc inspection showed that these are two of the most intensively cropped transects within the Canadian and US PPR respectively.

**Fig 6:**
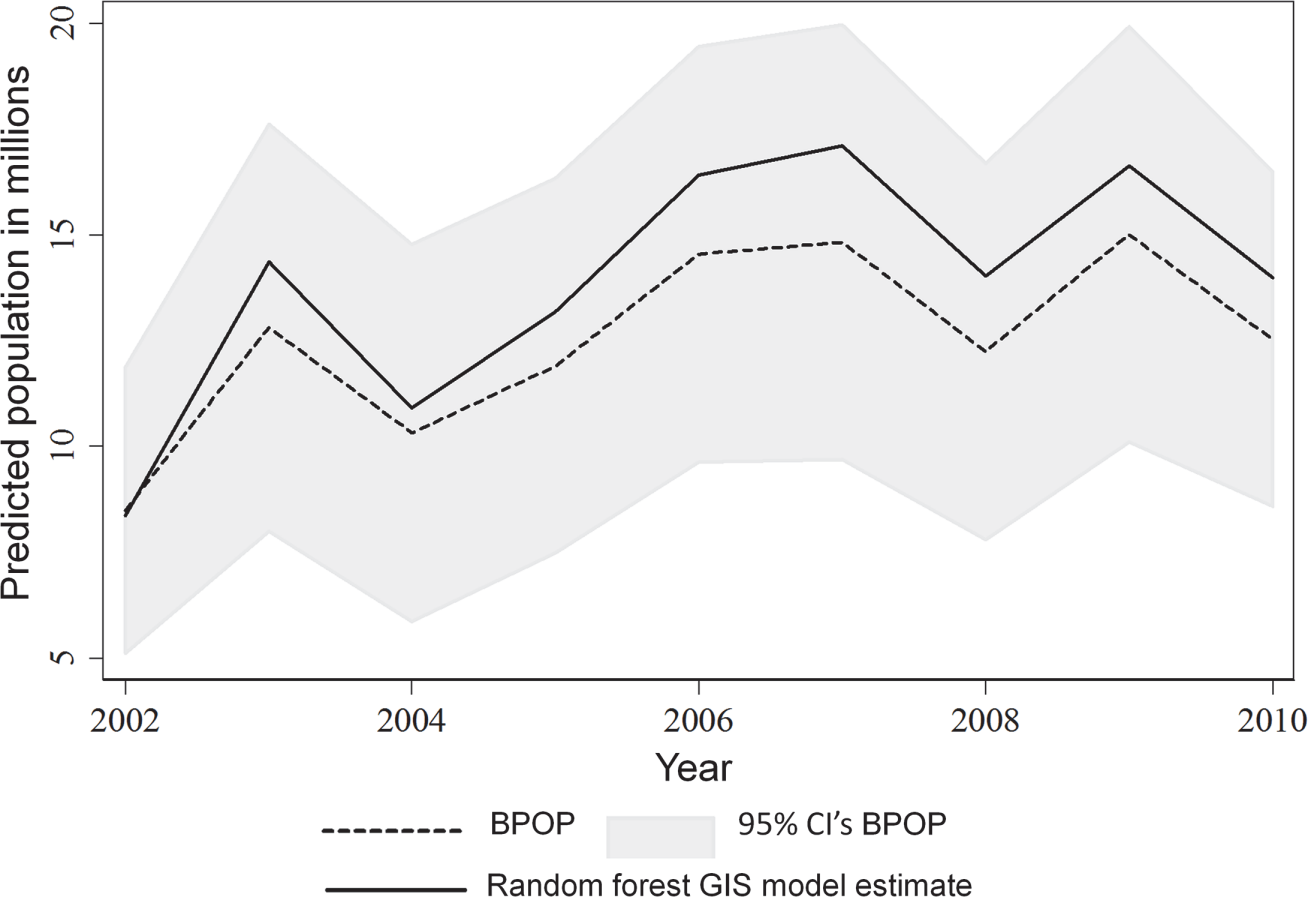
Comparison of design based BPOP estimates compared to population estimates generated by summing Random Forest predictions 2000–2002. We computed yearly 95% CI’s from transect and species-specific SE’s. We only compared BPOP versus Random Forest spatial methods for strata that had almost complete overlap (strata 26–28, 30, 32–35, 38–41, 45–47). For the years we modeled, summation of random forest spatial models across all overlapping strata predicted higher population estimates than the designed based BPOP estimates (Mean = 10.6% increase (range -1.6% [2002] to 15.3% [2007]), however estimates were within the 95% confidence intervals and population trends tracked each other.

Summation of our predictive spatial abundance models within a stratum and year predicted BPOP estimates generated by the USFWS-Division of Migratory Bird Management (Fig. 4). Using the summation of our predictive spatial abundance models within a stratum and year as the only predictor variable, produced an r^2^ of 0.977 and a regression coefficient which indicated a lack of bias in model fit (β = 1.005). We also graphically compared strata that had the highest duck densities during 2002–2010, and results indicate our spatial models and BPOP estimates tracked each other through time (Fig. 5, Fig. 6). However, we consistently over-predicted populations as estimated by BPOP methods in strata 34 & 47 (Fig. 5). Inspection of residuals in strata 34 and 47 showed these strata consistently had the highest standardized residuals across the 9 years we studied. Post hoc inspection of the habitat in these strata indicates they are two of the most intensively cropped regions within the Canadian and US PPR respectively. Across the entire U.S. and Canadian PPR we show predicted population trends tracked each other when graphically comparing estimates generated from the two different methods. We also show that spatially explicit models were within the 95% CI of BPOP estimates (Fig. 6) across all strata combined.

## Discussion

We were able to identify key areas for duck conservation across the PPJV & PHJV administrative areas using equivalent methodologies with high predictive capabilities (Tables 2 & 4, Figs. 2 & 4). Our models are the first seamless spatially explicit models of waterfowl abundance across the entire PPR and represent the initial step toward joint conservation planning between PPJV and PHJV. We did not explicitly define conservation thresholds within this paper, as explicitly defining conservation priority areas is inherently a partner driven process. However our waterfowl models do provide the methodological insight and create a quantitative link between the PPJV and PHJV allowing these groups to set joint waterfowl population and habitat objectives. Given asynchronous utilization of the U.S. and Canadian PPR by waterfowl populations during our study (Fig. 2), strong partnerships and joint planning capacity will be critical to achieving long-term goals.

While currently not incorporated into many conservation planning exercises, spatial measures of variability in population abundance could be important in framing conservation plans, especially for highly mobile animals such as waterfowl. Our predictive maps of the mean and SD of duck densities in conjunction indicate large differences in spatial and temporal abundances of ducks within smaller sub-regions in the PPR (Fig. 3). These differences have implications to both conservation planning and local-scale ecology. For example, in both countries certain landscapes consistently attracted higher numbers of waterfowl with low variability. Protection of these landscapes is important to ensure base population levels across a wide range of precipitation patterns. We also demonstrate wide variability in population distributions across the 9 years we studied (Figs. 2 & 3). Our work also draws attention to the likely increases in habitat area required to support defined population goals, when spatial and temporal variation are incorporated into conservation planning for highly mobile birds. This idea is consistent with recent theoretical research which documented increases in conservation areas are needed to offset population losses induced by increased environmental variability [70].

Incorporation of spatially explicitly knowledge of the mean and SD of duck densities may increase explained variation in waterfowl population and recruitment estimates. These are important parameters, because both are used in waterfowl harvest management models [71]. Recent work documented pulses in nest success with increases in nest success rates corresponding to pulses in primary productivity [41]. These pulses resulted from areas transitioning from a dry precipitation cycle to a wet precipitation cycle [27,72]. Understanding the location of pond counts in conjunction with status of the wet/dry precipitation cycles may be important to understanding recruitment. For example, relationships between pond counts and duck recruitment in areas such as northern South Dakota with high average abundance and high variability may differ from areas with lower SD estimates (Fig. 3). In areas like the north central portion of the Montana PPR, average densities generally were low across the 9 years we modeled. However when wetland conditions were favorable such as 2009, duck populations increased substantially (Fig. 2). Past work in this area documented higher recruitment compared to the core waterfowl breeding areas in the PPR [73]. If pulses in nest success with increases in nest success rates corresponding to pulses in primary productivity [41] are supported across a wide range of studies, it may be ecologically important in refining our understanding of the variation between pond counts, waterfowl breeding population estimates, and PPR-wide recruitment, all of which are important inputs into harvest management models [71]. Within our study, we documented a non-linear and positive association with higher Palmer Drought Severity Index Scores from prior years in most years (S1 File). This shows a pattern of waterfowl colonizing areas transitioning from drier to wetter states which has been shown to increase nest success rates [41].

**Fig 7:**
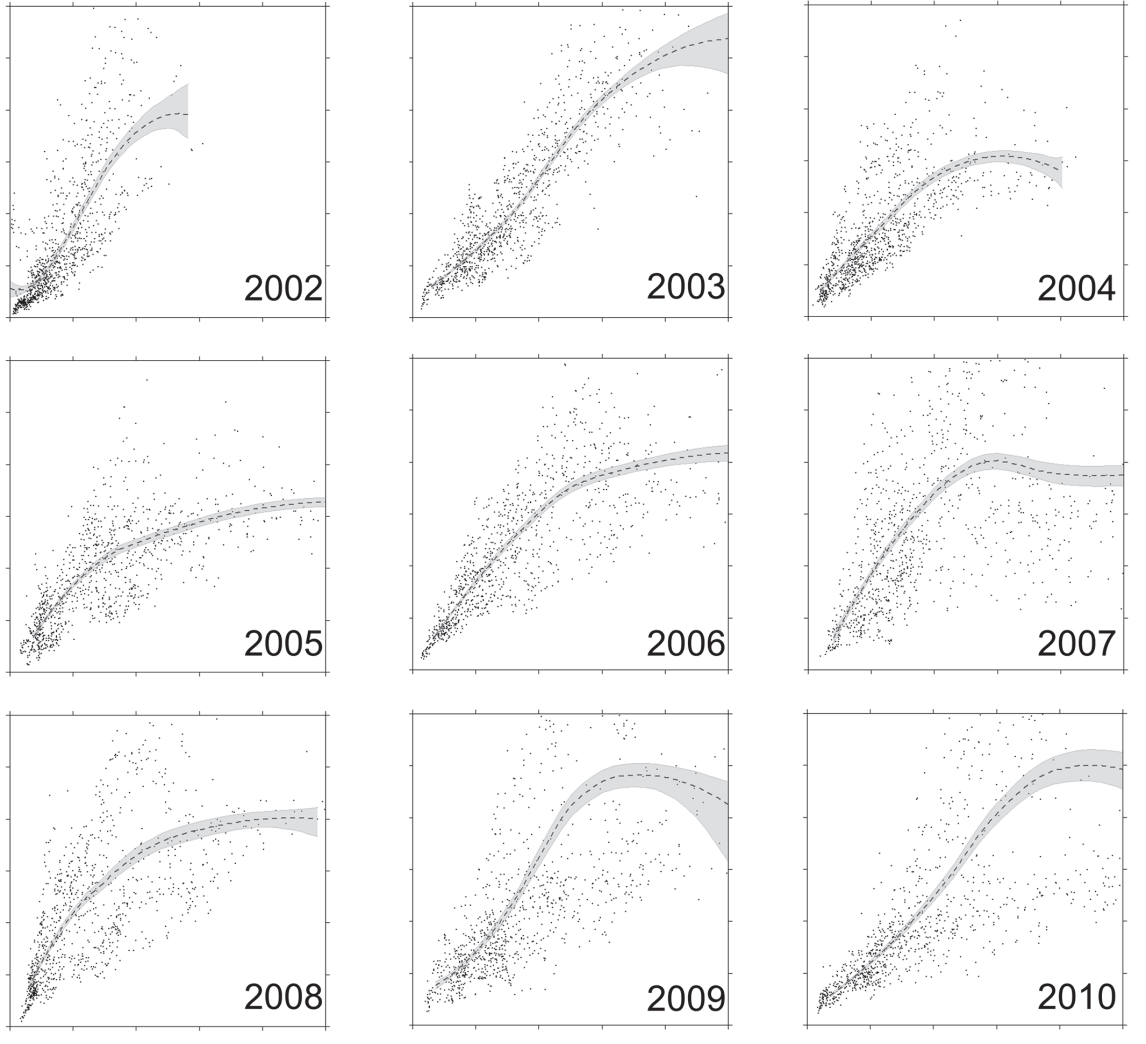
The functional response of waterfowl abundance to wetlands density varied with changing population sizes within the Prairie Pothole Region of the U.S. and Canada during 2002–2010. Waterfowl abundance was positively associated with wetlands density regardless of time lags tested, wetland density, or overall population size within the PPR. For each panel in the figure, the x-axis is the count of wetlands (0 to 100) and the y-axis is the count of 5 species of dabbling ducks (0 to 300) within a ∼ 11 km^2^ scale. Functional responses were generated using Loess smoothing functions in R.

We did not design this study to explicitly test hypotheses, none the less, we observed correlations between waterfowl settling and environmental conditions which could be used to generate and test additional hypotheses in the future. Not surprising, waterfowl were positively associated with wetland counts within the current year, or the previous 2 years. This was consistent regardless of the total waterfowl population or wetlands counts across the PPR. However, the functional relationship between waterfowl abundance and wetland counts changed as waterfowl populations increased from low to high population levels (Fig. 7). When populations were low, we document an almost linear relationship between waterfowl and wetlands, yet as populations increased the association between waterfowl and wetlands showed an asymptotic response (Fig. 7). At a minimum we could see two competing hypotheses for these relationships: 1) density dependence and 2) lagging population responses as the PPR transitioned from wet to dry conditions. Density dependence in waterfowl has been a topic of debate with some evidence supporting this idea and landscape scales [74,75], but see [76,77] for rebuttals. To date the mechanism which could cause continental recruitment measures to decline when duck populations are high have not been documented within field studies [75]. A second potential explanation for this relationship is simply wetland conditions change faster than waterfowl populations can respond. During 2002, pond counts were the lowest since the 80’s. Conversely waterfowl populations were near all-time documented highs in 2000 [32]. The ratio of waterfowl to available wetlands should be high in 2002 which could lead to the almost linear relationship detected in 2002. On the contrary, during 2009 duck populations were increasing from low population levels in 2002 [32]. The ratio of waterfowl to wetlands should be lower than in 2002 and could partly explain the asymptotic relationship. Explicitly designing a new experiment or analyzing data to try and tease these hypotheses apart is beyond the scope of this paper, but is worthy of future study.

Statistical model strength was higher than we initially expected for a pilot modeling effort, but we see areas for improvement in future analyses. Consideration of sub-segment population data is likely worthy and may increase predictive abilities in future analyses, especially for habitat selection questions involving individual choices. Recent research has derived equations allowing habitat selection models of individuals at sub-segment levels to be linked mathematically with our population level models hierarchically [78]. Building species-specific waterfowl models could also increase the amount of variation our abundance models explain by allowing them to tune to different life history strategies (e.g. Mallard vs. Pintails). Further, as harvest decisions are set on an individual species level, species-specific models will be needed to bring harvest and habitat managers together. Coordinated planning between countries was not possible with prior PHJV and PPJV models because they did not span the entire PPR ecosystem. However, one clear limitation of our pilot project is the lack of incorporation of Boreal Forest wetland habitats. Wetland habitats in the Boreal forest areas are known to be important to duck populations, and should be a priority to include in future efforts. Lastly, as uncertainties surrounding sub-PPR scale precipitation and temperature predictions are refined and have greater certainty [71], we feel planning for climate change will increase the importance of cross border planning.

The stratum level is a meaningful scale at which to compare population predictions generated by summing predictions of our random forest model within a stratum to BPOP estimates generated from a design-based sample and sample theory [21]. This is because waterfowl populations are estimated at the stratum level by the USFWS- Division of Migratory Bird Management and are then aggregated to generate species-specific population estimates. These population numbers are then used by the North American Waterfowl Management Plan to step down waterfowl population goals to specific joint ventures. We found high congruence between BPOP estimates and our spatial population model because we had both a high R^2^ value (0.97) and a slope that was not different from 1. Recognizing both methods use the same input data, we still maintain that population estimates generated from different methodologies, but reaching similar conclusions, create stronger inference about a population estimate than a single method [79].

A major goal of the current North American Waterfowl Management Plan and subsequent action plan is the linking of harvest and habitat management. Past work has developed an empirical and theoretical integration framework which could form the basis to link harvest and habitat management [71]. Recent work has derived a life-cycle metapopulation model which also conceptually begins linking harvest and habitat decisions for Northern Pintail [80]. These modeling frameworks represent large steps forward; however they rely on yield curves which are based on an aspatial mathematical population model [71] or a metapopulation model which parameterizes transition rates between large geographic areas which are much coarser than the scale of habitat conservation planning [80].

For migratory species like waterfowl, with the potential for distribution over a vast geographic range, decisions about where to settle and breed are known to have important demographic consequences at multiple scales. For example, spatial variation in reproductive success is evident at large scales within the breeding range of many North American ducks [47,81]. At local scales, reproductive success varies among specific available nesting habitats [82], and between cropland versus grassland-dominated landscapes [52,53]. Because waterfowl are well known to respond to annually varying environmental conditions [3], understanding and modeling how this variation drives settling within the PPR is a first step in accounting for the influence of multi-scale habitat selection on demographic rates at population scales [83,84].

We contend spatial aspects cannot be ignored if coherent harvest and habitat management decisions are to be made jointly. Spatially explicit models that incorporate landscape context into habitat prioritization and demographic response are the foundation on which habitat programs are currently delivered in the PPR. Conservation planning methods with the PPR are important because this area on average produces 50–75% of the primary game species of ducks, but only accounts for 10% of the waterfowl breeding habitats in North America [85]. Pond counts as currently used to represent habitat condition do not sufficiently represent the landscape context and spatial variation incorporated in PPJV and PHJV planning efforts. For example, we found pond counts during 2003 and 2005 were the most similar, but produced different spatial patterns in distributions of ducks across the PPR (Fig. 2). Aerial observers counted 31,341 and 30,769 ponds in 2003 and 2005 respectively, for a difference of 572 ponds. However, in 2003 and 2005 29% and 39% of the populations, respectively, were located in the parkland portion of the Canadian PPR. Furthermore, harvest models are based solely on Canadian pond counts. Because waterfowl productivity is heterogeneous across the PPR [47,52,81], our work demonstrates harvest models that overlook spatial and temporal variability in duck abundance, as well as landscape context embedded within the broad ecological gradients of the PPR, will likely lead to poorer predictions. Spatial population models used in conjunction with mathematical population models may allow better linkages and communication between harvest and habitat managers.

We believe our work forms the basis to begin joint international conservation planning across the entire PPR. We hope our work generates ideas on the possibility and potential of linking paradigms held by both population and habitat biologists. Above we outlined several ideas which may lead to progress in aligning conservation planning tools across the PPR as well as beginning to align harvest and habitat management. Our results shows at a minimum it is possible to produce spatially explicit waterfowl abundance models that produce similar stratum-level population estimates as design-based estimates currently used to set harvest regulations and NAWMP population goals for ducks. The fairly simple examples presented above highlight the conceivable importance of spatial heterogeneity and temporal variability in linkages between conservation planning and harvest management. We hope this effort generates discussion on the important linkages between spatial and temporal variation in population size, and distribution relative to habitat quantity and quality when linking habitat and population goals across this important region.

## Supporting Information

S1 File Final random forest predictive models and R code to allow interested readers to explore biological predictions and functional relationships in 3-D. (DOCX)
