# Supplementary material for: Building the Foundation for International Conservation Planning for Breeding Ducks across the U.S. and Canadian Border
Source: PLoS One. 2015 Feb 25;10(2):e0116735. doi: 10.1371/journal.pone.0116735 (PMC4340939; doi:10.1371/journal.pone.0116735)
Supplement: S1 File — (DOCX) [file pone.0116735.s001.docx]

**S1_File.docx**

**Final random forest predictive models and R code to allow interested readers to explore biological predictions and functional relationships in 3-D.**

We included the final random forest predictive models and R code to allow interested readers to explore biological predictions and functional relationships in 3-D. We did this because multi-variate kernel plots can be quite complex to interpret and do not lend themselves well to still graphics. Further, exploring variable responses without accounting for the strong effects of wetlands selection can mask relationships that could be otherwise evident.

**List of Workspace with Final Random Forest Model:**

If you wanted to simulate data through the final random forest model in 2002 you would load and label the model for use. General Syntax: Intduck_[YEAR].RData for the workspace in which the model is saved and Intduckdata_[YEAR].Rdata for the dataframe associated with the model. For all code you need to set the working directory to the folder in which the .RData is stored.

**Example Kernel Plot Code**

## loading the required programs, random forest model and data for an example in 2008

require(ks)

load("Intduck_2008.Rdata")

#TEST DATA is OK

load ("Intduckdata_2008.Rdata")

str(rf.data)

kde.data <- data.frame(Ducks=rf.data[,1], Ponds=rf.data[,11], PDSI=rf.data[,6])

str(kde.data)

# Create example kde estimate

H.scv <- Hscv(kde.data)

fhat <- kde(kde.data, H=H.scv)

plot(fhat)
